# Melanoma Biomarkers and Their Potential Application for In Vivo Diagnostic Imaging Modalities

**DOI:** 10.3390/ijms21249583

**Published:** 2020-12-16

**Authors:** Monica Hessler, Elmira Jalilian, Qiuyun Xu, Shriya Reddy, Luke Horton, Kenneth Elkin, Rayyan Manwar, Maria Tsoukas, Darius Mehregan, Kamran Avanaki

**Affiliations:** 1 Department of Biomedical Engineering, Wayne State University, Detroit, MI 48201, USA; monica.hessler@med.wayne.edu (M.H.); qiuyun.mri@wayne.edu (Q.X.); shriyar2003@gmail.com (S.R.); luke.horton2@med.wayne.edu (L.H.); kenneth.elkin@med.wayne.edu (K.E.); r.manwar@wayne.edu (R.M.); 2 Department of Dermatology, School of Medicine, Wayne State University School of Medicine, Detroit, MI 48201, USA; dmehregan@wayne.edu; 3 Department of Ophthalmology and Visual Sciences, University of Illinois at Chicago, Chicago, IL 60607, USA; elmira.jalilian@gmail.com; 4 Richard and Loan Hill Department of Bioengineering, University of Illinois at Chicago, Chicago, IL 60607, USA; 5 Department of Dermatology, University of Illinois at Chicago, Chicago, IL 60607, USA; tsoukasm@uic.edu

**Keywords:** melanoma, biomarkers, skin cancer, benign nevi, non-invasive

## Abstract

Melanoma is the deadliest form of skin cancer and remains a diagnostic challenge in the dermatology clinic. Several non-invasive imaging techniques have been developed to identify melanoma. The signal source in each of these modalities is based on the alteration of physical characteristics of the tissue from healthy/benign to melanoma. However, as these characteristics are not always sufficiently specific, the current imaging techniques are not adequate for use in the clinical setting. A more robust way of melanoma diagnosis is to “stain” or selectively target the suspect tissue with a melanoma biomarker attached to a contrast enhancer of one imaging modality. Here, we categorize and review known melanoma diagnostic biomarkers with the goal of guiding skin imaging experts to design an appropriate diagnostic tool for differentiating between melanoma and benign lesions with a high specificity and sensitivity.

## 1. Introduction

Melanoma is the fifth most common cancer type in the United States and is increasing in morbidity and mortality. Since 1975, the melanoma death rate has increased 16% while the incidence has quadrupled in both sexes, with male cases outnumbering female [1]. From 2007 to 2016, there has been a decrease in the incidence rate of 1.2% per year in individuals younger than 50 years of age, while the incidence rate is increasing by 2.2% per year for individuals >50 years old [2]. Although the average age of diagnosis is 63 and incidence increases with age, melanoma afflicts all demographics and is frequently diagnosed in individuals under the age of 30 [3]. The American Cancer Society anticipates 100,350 new cases of melanoma in 2020, with an estimated 6850 people ultimately succumbing to melanoma just this year [4].

**Figure 1:**
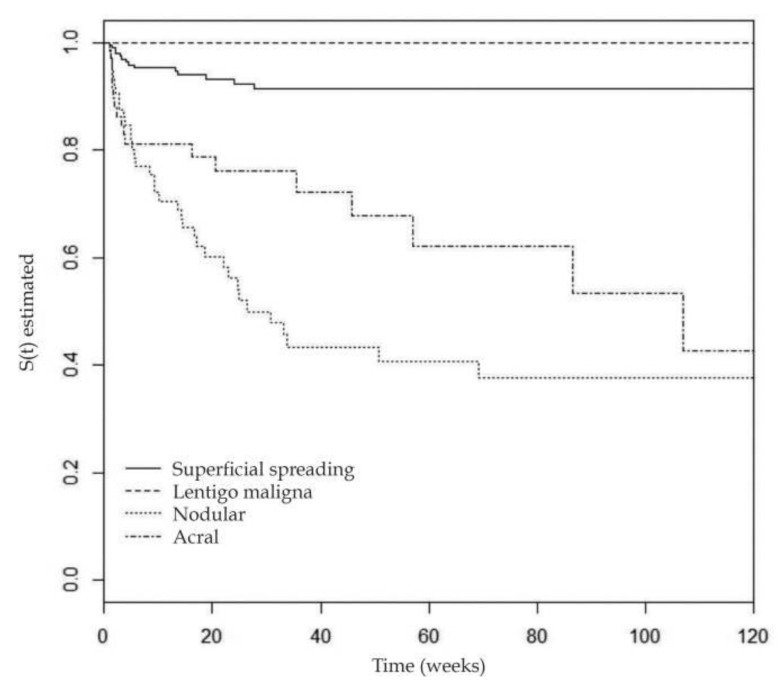
Estimated time to metastasis of melanoma subtypes (reproduced from “Prognostic factors for metastasis in cutaneous melanoma” by Cherobin et al. and is licensed under CC BY-NC-ND 3.0 from [7]). Kaplan–Meier curve showing the four different major subtypes of melanoma and the time (in weeks) for the development of metastasis in patients with primary cutaneous melanoma in the timespan of 1995–2012 [7]. This emphasizes the need for the early detection and diagnosis of melanoma.

Annually, estimates show that melanoma accounts for 65%–75% of skin cancer deaths despite comprising only 1% of all diagnosed skin cancers [3,4,5,6]. The thickness and invasion of the lesion in the skin is associated with metastatic capacity and these features are the most established predictors of mortality at diagnosis, rendering early diagnosis essential to patient prognosis. See Figure 1 for estimated time to metastasis of the different melanoma subtypes, emphasizing the importance of early and accurate diagnosis.

Patients with localized tumors (Breslow thickness < 0.8 mm) that undergo surgical excision can have a 5-year survival rate that is >97% [8]. The survival rate drops to 36% with nodal metastasis and to less than 10% for patients with distant metastasis [9]. There are four different major subtypes of melanoma including superficial spreading (60%–70%), nodular (15%–30%), lentigo maligna (10%), and acral lentiginous (5%) [10]. Other rare variants of melanoma include desmoplastic, spitzoid, melanoma within blue nevus or melanoma in a congenital nevus, pigmented epithelioid melanocytoma, and other variants.

**Figure 2:**
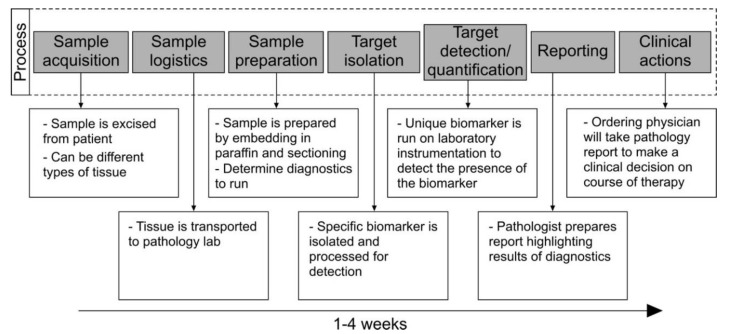
Melanoma diagnostic workflow (reproduced from [12]).

Although lesions can occur anywhere on the body involving both sun exposed and non-sun exposed areas, the most common locations are the head, neck, back, and lower extremities [11]. It is unsurprising that the most utilized diagnostic method for cutaneous melanoma is a full body skin exam by a specialist using the ABCDE criteria of asymmetry, border irregularity, color variation, diameter (>6 mm), and evolution, accompanied by dermoscopy and mole mapping techniques, and ultimately followed by biopsy and histopathologic analysis of suspicious lesions that appear different than other moles on the body [5]. Physical examination relies heavily on physician expertise and experience; therefore, efficacy is variable with a sensitivity of 57%–90% and a specificity of 59%–90% [11]. Currently, the most reliable method for the diagnosis of malignant melanoma is histopathology, which is obtained through biopsy techniques. Skin biopsy is more invasive and can be painful, traumatic, costly, and holds some risk of infection, scarring, or delayed healing at more challenging anatomic locations. Biopsy results also take time to get back to the patient, see Figure 2 for an overview of the biopsy process and timeline.

The consequences of missing a malignant melanoma are grave. As such, many biopsies are needlessly performed on clinically suspicious but still benign lesions to rule out melanoma. In fact, it has been shown that for every positive case of melanoma, there are 15 to 30 biopsies of lesions later proven to be benign [13]. Thus, the current method for melanoma detection has placed a significant economic burden on the healthcare system. At present, melanoma diagnosis is based on clinical examination and the ABCDE evaluation by specialists, followed by the selection of lesions that look different than the majority of existing moles on the body, dermoscopy and total body photography, excisional biopsy, and histopathologic examination by an expert dermatopathologist, and less often molecular analysis, genetic analysis, testing when indicated, and a multidisciplinary approach when indicated.

It is estimated that $32,594 dollars are spent for each melanoma detected [14]. Due to the increasing incidence of melanoma and the high cost of melanoma detection, there is a public health need for skin cancer screening with precise, cost-efficient methods. Particularly useful would be a non-invasive imaging technique to aid in melanoma diagnosis and the decision to biopsy.

**Figure 3:**
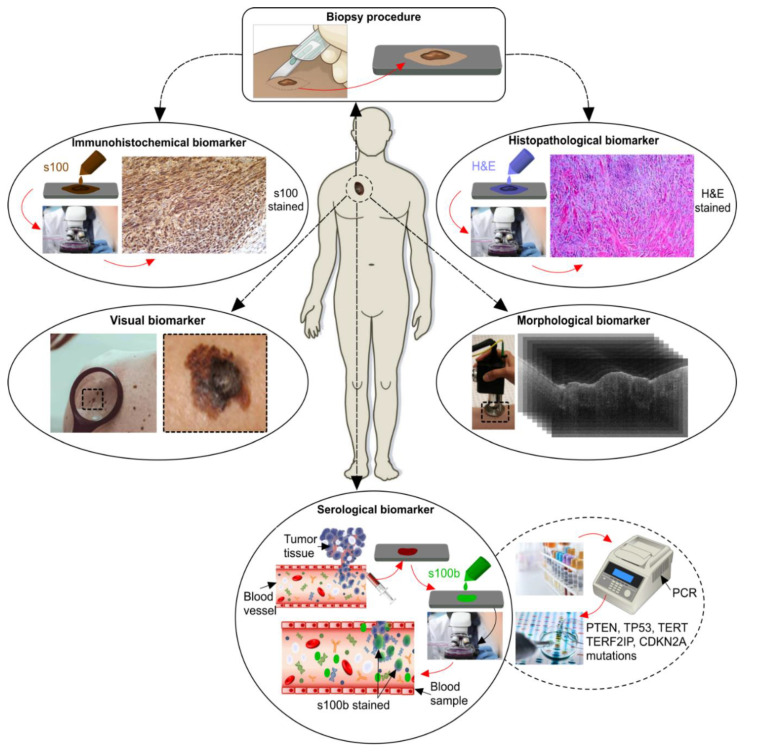
Melanoma diagnostic biomarkers (Immunohistochemical and histopathological images reproduced from “Desmoplastic Melanoma: A diagnostic dilemma” by Alva et al. under Creative Commons 4.0 International License (CC BY-NC-ND) from [15] and other images reproduced from [16].

Melanoma diagnostic biomarkers can be categorized into five categories including visual, histopathological, morphological, immunohistochemical, and serological/molecular biomarkers (see Figure 3). Visual biomarkers are the specific features of melanoma that dermatologists recognize on the patient with a naked eye or with the use of a dermatoscope. Histopathology of melanoma refers to the features that pathologists and dermatopathologists look for under the microscope after a biopsy of a suspicious lesion has been performed. The morphologic features of melanoma refer to the overall layer architecture and cellular structure of the lesions. Immunohistochemistry refers to a method of staining lesions for specific key markers, which aid in differentiating benign from malignant lesions. Lastly, serological/molecular markers refer to markers that can be detected in the peripheral blood or serum as indicators for melanoma.

## 2. Melanoma Progression

The transition from normal healthy skin to melanoma is a topic that has been studied and debated for years. Cutaneous melanoma originates from melanocytes located in the basal layer of the epidermis. Melanocytes comprise only 1% to 2% of epidermal cells but produce all of the melanin in the skin. Melanin production is stimulated by melanocyte stimulating hormone (MSH) released from keratinocytes via a p53-mediated mechanism in response to ultraviolet (UV) light [6,17].

There are two common types of melanin found in humans: (1) eumelanin—a brown-black pigmented melanin found in darker-skinned people, and (2) pheomelanin—a yellow-red pigmented melanin responsible for red hair and freckles [6]. Eumelanin has the ability to protect DNA more effectively than pheomelanin, absorbing more efficiently the harmful UV radiation and converting it to heat through a chemical process known as internal conversion (a process lacking in pheomelanin) [6]. This mechanism likely contributes to the higher incidence of skin cancer and melanoma observed in lighter-skinned individuals than in darker-skinned individuals.

Cells typically respond to UV radiation-induced DNA damage in one of two ways: the cell either repairs the DNA or initiates apoptosis (rarely they undergo necrosis or mitotic catastrophe) [18]. DNA is repaired by a number of cellular mechanisms including direct repair, nucleotide and base excision repair, and recombinational and cross-linked repair [19]. However, these mechanisms are error-prone processes that can potentially lead to the formation of mutations resulting in melanoma formation [6]. UVB radiation damages pyrimidines, leading to the formation of cyclobutene pyrimidine dimers and (6-4) photoproducts [20]. Repeated carcinogenic exposure from UV light results in an accumulation of mutations within the skin. Invasive melanoma contains a larger number of UV-related mutations compared to those found in benign nevi [21]. In addition, inherited conditions such as xeroderma pigmentosum (XP), congenital melanocytic nevi, familial atypical multiple moles and melanoma (FAMMM) syndrome, and *BRCA2* mutation all provide evidence for a genetic predisposition to the development of melanoma [22,23].

Unlike non-melanoma skin cancers (NMSC), melanoma can develop in areas that rarely receive sun exposure, such as the palmar surfaces of the hands and feet, and mucosal surfaces [24]. These melanomas are understood to have distinct oncogenic mutations uncommon in melanomas in areas of chronic ultraviolet (UV) exposure. One study found that melanomas located in areas of minimal sun exposure commonly displayed mutations in *BRAF* or *NRAS,* while melanomas in chronically sun exposed areas are most commonly associated with mutations in *TP53*, evidencing that melanoma is a heterogeneous disease stemming from genetic risk factors and accumulated environmental exposures [24,25].

**Figure 4:**
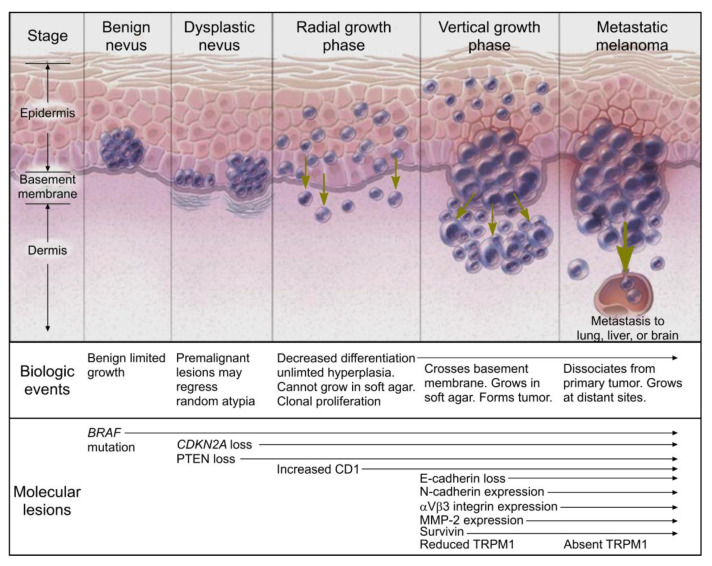
Melanoma progression diagram (reproduced with permission from the New England Journal of Medicine, Arlo J. Miller & Martin C. Mihm, “Melanoma,” 355: 51–65, Copyright © 2020 Massachusetts Medical Society (Waltham, MA, USA). Reprinted with permission from Massachusetts Medical Society, [32]). Benign nevi have been known to express *BRAF* mutations that allows for benign and limited growth. This mutation results in the activation of mitogen-activated protein pathway. In the evolution to a dysplastic nevus, cytologic atypia becomes more prevalent through the loss of cyclin dependent kinase inhibitor 2A (*CDKN2A*) and phosphatase and tensin homologue (PTEN), resulting in what is often referred to as a premalignant lesion. The radial growth phase shows clonal proliferation and decreased differentiation accompanied by decreased expression of MITF (micropthalmia-associated transcription factor). The formation of a tumor characterizes the vertical growth phase with the crossing of the basement membrane. An absence of TRPM1 (melanocyte-specific gene melastitin 1) correlates with metastatic capability, but the function of the gene is unknown. There are several other genes involved in the melanoma including loss of E-cadherin, increased expression of N-cadherin, αVβ3 integrin expression, and survivin.

Recently, the thought of a simple linear progression from nevus to melanoma in situ does not appear to occur [21]; rather, it is the result of an accumulation of multiple different mutations [26]. It has been found that melanoma associated mutations can be either somatic or due to environmental factors that are acquired over time [12]. As illustrated in Figure 4, in order to transform from benign nevus to melanoma, multiple mutations or “hits” must occur. Tsao et al. found that there is a 0.03% (men) and 0.009% (women) lifetime risk of a mole that is present by age 20 to later transform into cutaneous melanoma by age 80 [27]. In the work by Bastian, he suggests that there is an inciting oncogenic event that is often a gain of function mutation involving one of the following: *NRAS*, *HRAS*, *BRAF*, *KIT*, *GNAQ*, *GNA11*, *ALK*, *ROS1*, *RET*, and *NTRK1* [26]. Given that 30% of cutaneous melanoma arise near a nevus, often with the *BRAF^V600E^* mutation [28], the initial oncogenic mutation is helpful in separating different lesions such as congenital nevi, pigmented lesions on chronic sun damaged (CSD) skin, non-CSD skin pigmented lesions, spitz tumors, and blue nevi [26]. Secondary and tertiary oncogenic events usually involve a loss of tumor suppressor genes such as *CDKN2A*, *TP53*, *PTEN*, or *BAP1*, and these can be used for determining disease progression within classes [26].

Melanomas can be sorted into two categories based on the skin on which they arise: CSD and non-CSD. CSD melanomas develop on skin showing solar elastosis, deterioration of the dermal elastic fibers, and they are often found in individuals >55 years old after years of UV radiation often on the head and neck, while the non-CSD melanomas usually affect individuals <55 years old in areas with intermittent sun exposure such as the trunk [29]. Non-CSD melanomas are often superficial spreading melanomas that can develop within a previous nevi in younger patients [30]. Non-CSD melanomas are often associated with *BRAF^V600E^* mutations that are found in common nevi as well, while CSD melanomas are often seen to have *NF1*, *NRAS*, or *BRAF^nonV600E^* mutations [29,31].

**Figure 5:**
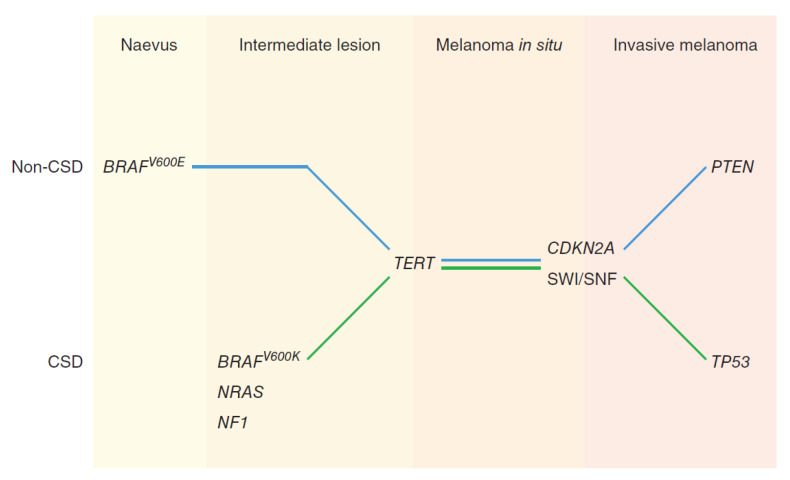
Melanoma Progression on Chronic Sun Damaged (CSD) versus Non-CSD Skin: (Reproduced from “Melanoma” by Guterres et al. with permission from the publisher John Wiley and Sons, Wiley Online Library: Copyright © 2020 John Wiley & Sons. Ltd. (Hoboken, NJ, USA) [31]). Melanoma progression can be separated into the two pathways of Non-CSD (non-chronically sun damaged) skin and CSD (chronically sun damaged) skin melanomas. Non-CSD melanomas often have a mutation in *BRAFV600E* with a resultant benign nevus. In contrast, CSD skin forms intermediate dysplastic lesions after mutations occur in *Non-BRAFV600E (BRAFV600K)*, *NF1*, and *NRAS*. Resultant *TERT* reactivation leads to progression to early melanoma for both Non-CSD and CSD skin. If the lesions acquire further loss of function mutations in *CDKN2A* or *SWI/SNF*, the lesion is keyed up for invasion. Lastly, *PTEN* (more common in *BRAFV600E* melanomas) inactivation or TP53 (*NRAS* and *NF1* melanomas) inactivation leads to more invasive melanomas [31].

Within a nevus, limited proliferation occurs due to the initiating mutation. If additional mutations are acquired such as *TERT* promoter mutations on both non-CSD and CSD skin, this results in further proliferation toward melanoma [31]. The characteristic histologic pagetoid growth pattern is associated with non-CSD melanoma with *BRAF^V600E^* mutations [29]. In contrast, melanocytes with high cumulative sun exposure can result in the formation of lentigo maligna with its characteristic lentiginous growth pattern that can cover several centimeters of skin for years before generating a nodule and becoming invasive, making it more common in older individuals with years of sun damage [29]. Ultimately, loss of function in *CDKN2A* or *SWI/SNF* primes lesions to become invasive, with mutations in *PTEN* and *TP53* promoting complete invasion [31]. These mutation pathways are illustrated in Figure 5. As found in the study by Colebatch et al., a simple linear progression from nevus to invasive melanoma does not appear to occur, but instead, different branches of mutations occur later in the progression of melanoma with a resultant heterogeneity of neoplasms [21].

## 3. Method

In order to select the articles related to current diagnostic biomarkers, the initial search was done using the PubMed database on 15 April 2020 by searching melanoma diagnosis and detection as a MeSH Major Topic. This search without filters yielded 163,271 results. Additional filters of full text, clinical trials, meta-analysis, randomized control trial, review, and systematic review were applied. Choosing English as the language, human subjects, and the subject cancer narrowed our search down to 116 results. Each abstract was examined for relevance to our search topic of melanoma biomarkers. Of the 116 abstracts, 11 papers were selected out for closer examination. An additional PubMed search was completed under the terms melanoma diagnosis biomarkers with the same filters of full text, clinical trials, meta-analysis, randomized control trial, review, and systematic review applied, yielding 828 results. Eleven additional studies were selected out from the results. One final search was completed on Google Scholar using the following search terms of melanoma diagnosis biomarkers, OR histopathology, OR pathology, OR visual OR inspection, OR serum OR biomarker melanoma, which resulted in 10,500 results. The search was further stratified to include the “allintitle” feature with the search terms, melanoma diagnosis biomarkers OR histopathology OR pathology OR visual OR inspection OR serum OR biomarker OR pathology yielding 93 results. The abstracts were reviewed, duplicates were removed, and citations unable to be accessed in full text were also removed. Six of the articles were chosen for further review of the entire paper. In order to create the most comprehensive review, we also examined the references of the original articles that came up in the search to fill in any missing areas. The inclusion criteria were set to include review papers, randomized controlled trials, and meta-analyses aimed at addressing diagnostic biomarkers for melanoma. Exclusion criteria included papers and studies focused on prognostic biomarkers, melanoma therapy, or melanoma staging, as these types of papers drew away from the aim of our study, which was focused on compiling the current most clinically utilized diagnostic biomarkers of melanoma.

## 4. Melanoma Biomarkers

### 4.1. Visual

**Figure 6:**
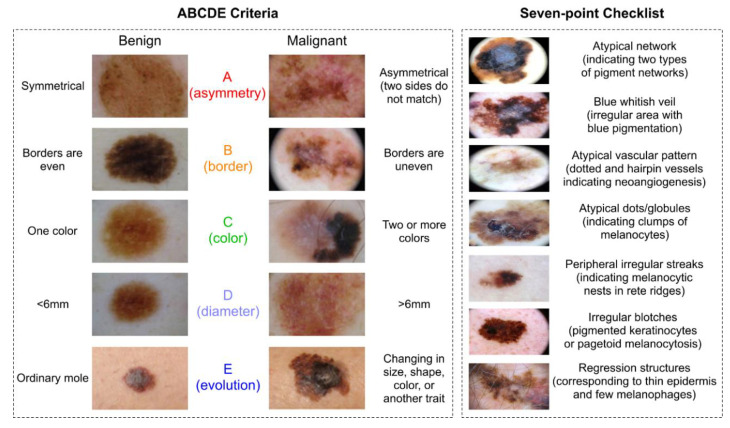
Visual melanoma biomarkers: ABCDE criteria of asymmetry, border irregularity, color variation, diameter (>6 mm), and evolution, reproduced from [37]. Seven-point checklist images (1st and 4th are reproduced from “Dermoscopy of Melanomas on the Trunk and Extremities in Asians” by Mun et al. under the Creative Commons Attribution License from PLoS ONE, Copyright © 2020 Mun et al. [38]; images, 2nd, 5th, 6th, are reproduced from “Dermoscopic characteristics of melanoma according to the criteria ‘ulceration’ and ‘mitotic rate’ of the AJCC staging system for melanoma” by Deinlein et al. under the Creative Commons Attribution License from PLoS ONE, Copyright © 2020 Deinlein et al. [39]; 3rd image is reproduced from “Dermoscopy and pigmented lesions of oral cavity” by Bajpai et al. under the Creative Commons Attribution 4.0 International License in the Ankara Medical Journal by Ankara Yildirim Beyazit University [40]; 7th image is reproduced from “Lentigo Maligna: Clinical Presentation and Appropriate Management” by Iznardo et al. under the Creative Commons Attribution-Non-Commercial (Unported v3.0) License from Dove Medical Press Limited (Macclesfield, UK) [41].

Differentiating a benign nevus from cutaneous melanoma is first done through visual inspection. Visual criteria for melanoma detection include the ABCDE criteria of asymmetry, border irregularity, color variation, diameter (>6 mm), and evolution, with “E” being officially added in 2004 [33]. These features are demonstrated in the left side of Figure 6. Thomas et al. found that using two criteria in combination leads to sensitivity of 89.3% and specificity of 65.3%, while utilizing three criteria brings sensitivity to 65.55% and specificity to 80% [11,34]. Identifying visual features can be difficult with lesions that are not pigmented such as nodular amelanotic melanoma [34]. Dermoscopy or dermatoscopy is a method of examining the skin using skin surface microscopy. Russo et al. presented a seven-point checklist of melanoma used in dermoscopy including (I) atypical network (indicating two types of pigment networks), (II) blue whitish veil (irregular area with blue pigmentation), (III) atypical vascular pattern (dotted and hairpin vessels indicating neoangiogenesis), (IV) atypical dots/globules (indicating clumps of melanocytes), (V) irregular streaks (indicating melanocytic nests in rete ridges), (VI) irregular blotches (pigmented keratinocytes or pagetoid melanocytosis), and (VII) regression structures (corresponding to thin epidermis and few melanophages) [35]. In dermoscopy of acral lesions, benign lesions often show a parallel furrow pattern (linear pigmentation in furrows of the sole) in comparison to malignant lesions with parallel ridge pattern (parallel band-like pigmentation in ridges of the sole (gold standard for diagnosing volar melanocytic nevus and malignant melanoma) [36]. The ABCDE criteria and the seven-point checklist visual biomarkers are summarized in Figure 6.

### 4.2. Histopathology

**Figure 7:**
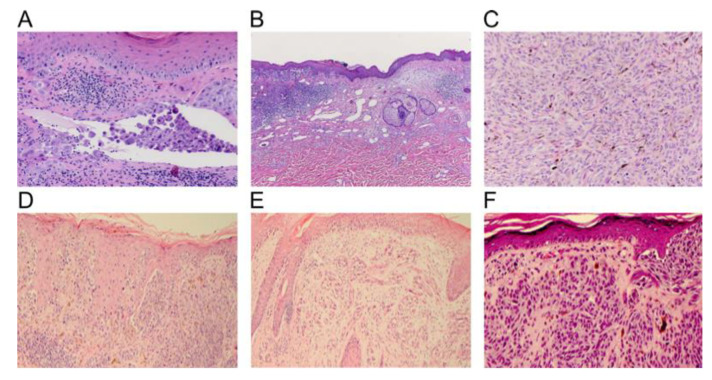
Histopathological melanoma features. (**A**, **B**, and **C** reproduced from “Progress in melanoma histopathology and diagnosis” by Piris et al. with permission from the publisher Elsevier, Copyright © 2020 Elsevier Inc. (Amsterdam, The Netherlands) All rights reserved [45]) (**A**) Vascular invasion of malignant cells, (**B**) Lower-power view demonstrating partial regression of malignant melanoma, (**C**) dermal mitotic figures with dark blue pycnotic nuclei [45], (**D**–**F**) reprinted with permission from “The classification of cutaneous melanoma” by LM Duncan with permission from the publisher Elsevier, Copyright © 2020 Elsevier Inc. All rights reserved [46]. (**D**) Intraepidermal tumor cells within a superficial spreading melanoma with nests of cells and individual cell scatter present. (**E**) Intraepidermal tumor cells in a lentigo maligna melanoma that are located at the base of the epidermis with extension down the follicle of hair, and with the presence of epidermal atrophy. (**F**) Nodular melanoma with minimal tumor within the epidermis with nested proliferations of melanoma cells [46].

While visual examination is limited to the horizontal plane of view (surface of the lesion), the next logical step is to examine the lesion in the vertical plane [42]. This is done by either a pathologist or dermatopathologist who analyzes the biopsied specimen stained with hematoxylin–eosin (H&E) staining to allow for the visualization of structures from the epidermis through the reticular dermis and subcutaneous tissues [43]. Criteria for the diagnosis of melanoma includes overall asymmetry and poor circumscription, poor or variably sized nests, single cells predominating over nests, upward scatter of melanocytes and nuclear pleomorphism, and morphologic changes of the nucleus and cytoplasm. Pathologists do have a set of mandatory histopathological qualities of melanoma (see Table 1) that must be included in the pathology report of a melanoma including ulceration, mitotic rate, regression, lymphovascular invasion, perineural invasion, Breslow thickness, satellitosis, and status of surgical margins [44]. Figure 7 demonstrates the histopathology features that histopathologists often use to diagnose melanoma.

**Figure 8:**
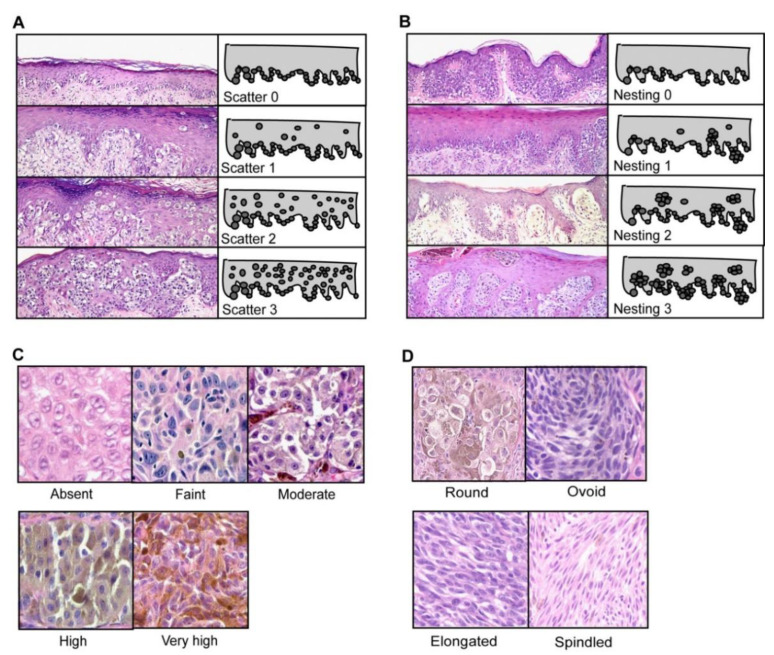
Grading of cellular morphological features (Reproduced from “Improving melanoma classification by integrating genetic and morphologic features” by Viros et al. under Creative Commons Attribution License from PLOS Med, © Viros 2008 et al. [47]). (**A**) Shows grading of scatter of intraepidermal melanocytes from 0 to 3, increasing in scatter. Grade 0 shows all melanocytes along the dermal–epidermal junction; grade 1 shows >75% of melanocytes along the dermo-epidermal junction, with some present higher in the epidermis; Grade 2 showed equal amounts of intraepidermal melanocytes at the junction and higher in the epidermis; Grade 3 is noted when >50% of intraepidermal melanocytes are in the upper epidermis, (**B**) shows the grading of nesting of intraepidermal melanocytes from 0 to 3. The amount of nesting is quantified here as: Grade 0: Intraepidermal melanocytes present as single cells with rare nests; Grade 1: Intraepidermal melanocytes arranged as single cells with <25% of cells in nests; Grade 2 Intraepidermal melanocytes in nests in 25%–50%; Grade 3: >50% of intraepidermal melanocyte population are arranged in nests, (**C**) Cytoplasmic pigmentation of neoplastic melanocytes, Scaled 0–4. Score of 0 meant no pigmentation is present; Score 1: Faint pigmentation barely visible at low power; Score 2: Moderate pigmentation visualized at low power; Score 3: High pigmentation easily visible at low power, pigmentation of cytoplasm is similar to the nucleus pigmentation intensity; Score 4: Very highly pigmented cytoplasm, often obscuring nuclei [47]. (**D**) Cell shapes were also graded 0–3, Grade 0: Round cell with equal diameter and length; Grade 1 was ovoid with a diameter 1/3 longer than the short diameter; Grade 2 is elongated with a long diameter 1/3–2 times longer than the short diameter; Grade 3 is spindled with a long diameter two times the shorter diameter [47].

Viros et al. also examined the cellular morphologic features of melanoma that included the scatter and nesting of intraepidermal melanocytes, cytoplasmic pigmentation of neoplastic melanocytes, cell and nuclei shapes, size, and epidermal contour. Examining the scatter of intraepidermal melanocytes means tracing the location of melanocytes within the epidermal layers and comparing it to their normal presence along the dermo-epidermal junction (DEJ). Viros et al. classified the degree of nesting as a location of five or more melanocytic cells together. Pigmentation was scaled according to maximum pigmentation anywhere in the tumor, the average pigmentation across all sections in radial growth phase, and average pigmentation of a section of the vertical growth phase. Cell shape and size can be examined microscopically, and lymphocytes (usual diameter of 4–5 μm) serve as a control for size reference. Cell shape can be classified by ovoid, elongated, or spindled. Epidermal contour in the radial growth phase can range from atrophic to hyperplastic and increases with tumor thickness [47]. For examples of these features in histopathology, please see Figure 8.

According to the study done by Elmore et al., 82.5% (81%–84.5%) of diagnostic skin biopsies of melanocytic skin lesions are further confirmed with a reference panel of knowledgeable pathologists with 9.2% (8.8%–9.6%) of cases being under-interpreted and 8.0% (6.2%–9.9%) of cases overinterpreted [48]. This inter and intra-observer variability has been noted by other authors such as Davis et al., and it is seen primarily in lesions with indistinct features on histopathology [49]. With these studies, it is evident that the diagnosis of melanoma requires a multistep process with room for improvement.

### 4.3. Morphology

**Figure 9:**
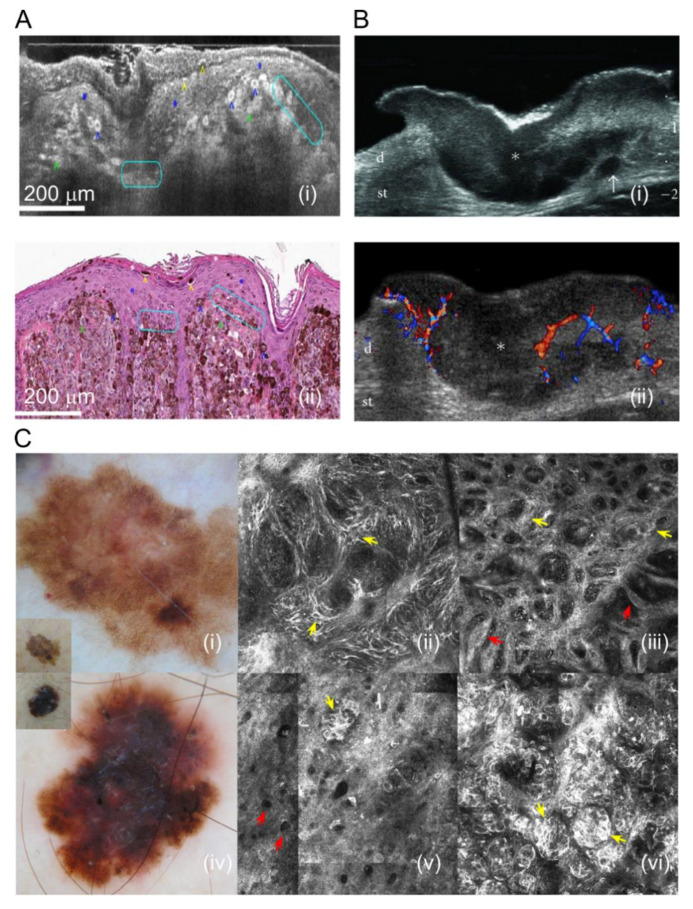
Morphological melanoma features (**A**) (Reproduced from “Line field confocal optical coherence tomography for high-resolution noninvasive imaging of skin tumors”, doi:10.1117/1.Jbo.23.10.106007 by Dubois et al., published by SPIE, reproduced under Creative Commons Attribution 3.0 Unported License from [91], © The Authors). LC-OCT (Line field-optical coherence tomography) image of cutaneous melanoma (**i**) and a histopathology slide of the corresponding lesion (**ii**). Blue star: epidermis; white star: stratum corneum; blue arrowhead: pagetoid spread of tumor epithelial cells; yellow arrowhead: tumor cells being eliminated; green arrowhead: clumps of melanocytic tumor cells in the dermis; turquoise circles: partial DEJ (dermal–epidermal junction disruption) [91]. (**B**) (Reproduced from “Sonography of the primary cutaneous melanoma: a review” by Wortsman under the Creative Commons Attribution License [92]) Transverse ultrasound image (**i**) of melanoma on the abdominal wall demonstrating fusiform hypoechoic lesion (*) that is invading the dermis and subcutaneous tissue. The arrow is pointing to a satellite metastasis. Doppler ultrasound (**ii**) shows increased vasculature and blood flow. Abbreviations: d: dermis; st: subcutaneous tissue [92]. (**C**) (Reproduced from “Reflectance confocal microscopy features of BRAF V600E mutated thin melanomas detected by immunohistochemistry” by Urvanegia et al. under Creative Commons Attribution License from PLOS One, © 2020 Urvanegia [93]). Reflectance confocal microscopy images are demonstrated in panels (**ii**), (**iii**), (**v**), and (**vi**). (**i**) is a dermoscopy image of a superficial spreading melanoma with a broadened pigment network. (**ii**) (1.5 × 1.5 mm) illustrates singular atypical cells (yellow arrows) causing dermal–epidermal thickening. (**iii**) (1.5 × 1.5 mm) shows a meshwork pattern (red arrows), thick interpapillary spaces (yellow arrows), and nonedged papillae at the dermal–epidermal junction. (**iv**) is a dermoscopy image of superficial spreading melanoma with a multicomponent pattern. (**v**) (0.75 × 0.75 mm) shows epidermal nests and hyporeflective pagetoid cells in the epidermis (red arrows). (**vi**) (0.75 × 0.75 mm) demonstrates dermal–epidermal nests (yellow arrows) [93].

Morphologic features can be examined through different non-invasive imaging modalities including Optical Coherence Tomography (OCT), Reflectance Confocal Microscopy (RCT), and Ultrasonography see the complete list of these imaging modalities in [43,50,51,52,53], including quantitative dynamic infrared imaging, hyperspectral imaging, multispectral imaging, electrical impedance spectroscopy, and photoacoustic imaging (both microscopy and tomography) [54,55,56,57,58,59]. Raman spectrometry, real-time elastography, terahertz pulse imaging, multiphoton imaging, magnetic resonance imaging, positron emission tomography, fiber diffraction, Fourier transform infrared spectroscopy, and reflex transmission imaging. Examples of OCT, RCT, and ultrasonography images of melanoma are shown in Figure 9. It should be noted that many of these imaging modalities are in the investigational phase (see [60,61,62,63,64,65,66,67,68,69,70,71,72,73,74,75,76,77,78,79,80,81,82,83,84]); i.e., they are not regularly used in clinical practice as of yet, but they could provide promising options in the future when they are better understood and accessible.

Rajabi-Estarabadi et al. reviewed the literature on the use of OCT to detect morphologic features of melanoma. These features included architectural disarray, stromal reaction, atypical melanocytes, vertical location of atypical melanocytes, pagetoid spread, junctional nests, and dermal nests [84]. Vessel morphology can also be examined through the use of speckle variance optical coherence tomography (SV-OCT) to detect the irregular organization of vessels found in melanoma [84].

Reflectance Confocal Microscopy (RCT) is another non-invasive method to study skin cancer in vivo. RCT also allows for visualization of the tissue microstructure in tumorous lesions. Morphologic biomarkers such as pagetoid melanocytes can be detected by RCT [85]. Other morphologic features detected by RCT are broken down by skin layers by Waddell et al. [86]. In the superficial epidermis, atypical honeycomb pattern, atypical cobblestone pattern, and pagetoid cells are often seen in melanomas. In the basal cell layer and the dermo-epidermal layer (DEJ), cellular atypia, nonedged dermal papillae, and a disarranged DEJ can be appreciated. Lastly, the upper dermis can have cells distributed in sheet-like structures and sparse nests composed of round or pleomorphic cells [86].

High-frequency ultrasound (HFUS) has also been utilized in the diagnosis of melanoma [87]. Dinnes et al. found in their analysis that melanotic lesions appear hypoechoic, homogenous, and well defined on ultrasound [87]. Doppler ultrasound can be utilized to assess tumor vascularity by characterizing vascularization and the number of vascular pedicles present [88]. Giovagnorio et al. found that hypervascularity had a sensitivity of 90% and specificity of 100% in contrast to the benign lesions that showed hypovascularity with a sensitivity of 100% and specificity of 90% [89]. Strain elastography can be utilized to assess tumor stiffness, which is likely due to increased cellularity and tumor infiltration, as noted by Botar et al. [88].

One of the parameters that can be well studied using the above-mentioned modalities is the depth of the tumor in skin. The depth of tumor invasion correlates with the thickness of the tumor, which is strongly related to prognosis. Thickness of the tumor assists in the staging of the melanoma, which is based off of Breslow’s Depth, which was updated in 2017 in the 8th Edition of the AJCC Cancer Staging Manual. The depth is measured from the epidermal granular level to the deepest level of invasion [90]. The stages are as follows T1: ≤1.0 mm, T1a: <0.8 mm with no ulceration, T1b: 0.8–1.0 mm with or without ulceration or <0.8 mm with ulceration, T2: 1.01–2.0 mm, T3: 2.01–4.0 mm, and T4: >4.0 mm. Additionally, mitotic rate is no longer in the T category [90].

### 4.4. Immunohistochemical Stains

When the limitations of histologic examination are reached, special stains and immunohistochemical analysis provides tools to differentiate malignant lesions from benign nevi. Multiple targets have been noted in several reviews including but not limited to S100, Gp100, Anti-MART-2, Anti Melan-A, CSPG4 (Chondroitin Sulfate Proteoglycan 4), pHH3, and p16 [45,94,95]. A comprehensive list of immunohistochemical biomarkers is given in the review by Abbas et al. [95]. As noted by Eisenstein et al., these biomarkers indicate the existence of melanoma, as opposed to separating it from other cancer types [94]. While there are many immunohistochemical markers that are currently known, we have focused on several of the most clinically utilized markers, as well as several markers that are utilized for the discernment of ambiguous lesions. These biomarkers include S100, HMB 45, Ki-67, Melan A (MART1-Melanoma antigen recognized by T cells 1), Chondroitin Sulfate Proteoglycan 4 (CSPG4), Tyrosinase, PNL2, MITF (Microphthalmia transcription factor), SOX10, MC1R (Melanocortin 1 Receptor), PRAME (preferential expressed antigen in melanoma), pHH3, and p16.

S100 is a protein family with at least 25 identified members encoded by many genes, but most are located on chromosome 1q21 in a region called the epidermal differentiation cluster. These proteins have a known expression in melanoma [96]. S100 is involved in multiple cellular processes including cellular growth, cell cycle progression, cellular motility, calcium homeostasis, transcription, and protein phosphorylation [97,98]. Eisenstein et al. reported 90% sensitivity in the immunohistochemistry (IHC) stain of S100 in primary and metastatic lesions of melanoma [94]. This is in agreement with the work done by Nonaka et al., finding that S100 is the most sensitive marker for melanoma, particularly with the subtypes S100A1, S100A6, and S100B [99]. In their study [99], more than 90% of the malignant melanomas were found to express these proteins; S100A1 specifically was present in all types of melanomas but was not present in neurofibromas, schwannomas, or malignant peripheral nerve sheath tumors [97,99]. In contrast, S100A6 was strongly and diffusely positive in the junctional and dermal components of 100% (42/42) studied spitz nevi, positive in 56% melanocytic nevi (41/73), but only positive in 33% (35/105) of the dermal components of melanomas in the study done by Ribé and McNutt [100]; therefore, they proposed the idea of utilizing S100A6 for the differentiation of Spitz nevus from melanoma [100].

HMB 45 is a monoclonal antibody against PMEL17, which is also called gp100 and plays a role in the organizational structure of melanoma [97,101]. While it can stain positively in nevi, the stain is usually limited to the epidermal and papillary dermal melanocytes in benign nevi [102], while in primary melanoma, the staining pattern is in both the superficial and the deep melanocytes of the lesion [97]. HMB 45 could be particularly useful in combination with Ki-67 [97,102,103], which is discussed as an additional marker in this manuscript. In the past, there was discussion in regard to false positive results in other forms of cancer, but currently, Ordóñez states that other tumors such as epithelial, lymphoid, glial, and mesenchymal origin tumors are negative [97]. However, HMB 45 can be seen in other tumors such as angiomyolipoma, lymphangiomyomatosis, and the clear cell “sugar” tumor, and it has also been seen to be positive in post inflammatory hyperpigmentation, making it less reliable as a melanoma marker according to the review by Eisenstein et al. [45,94].

Ki-67 is a non-histone nuclear protein and is useful as a marker of proliferation. Due to the detection of Ki-67 in all cell cycle phases except in the resting phase G_0_, Ki-67 is thought to be a more useful marker of proliferation than mitotic rate [95]. Ki-67 is found to be positive in <5% of common nevi, while being positive in 13%–30% of melanoma tumor cells, with cases showing 100% nuclear positivity [95,104,105].

Melan A, also known as MART 1 (Melanoma antigen recognized by T cells-Cloned gene) [106] is found in both melanosomes and the endoplasmic reticulum, which aids in the processing and transportation of PMEL (premelanosome protein). PMEL is a key factor in the creation of melanosomes [107]. Rochaix et al. stated that “Immunohistochemical studies have shown Melan A expression in all (100%) dysplastic, junctional, intradermal, compound, Spitz, and congenital nevi, as well as in lymph node capsular nevi.” [108]. Melan A is a highly sensitive marker that is not expressed in the dendritic cells of lymph nodes like S100 is, which makes Melan A an appropriate candidate for melanoma detection in lymph nodes. Melan A is also not expressed in histiocytes and is reported to be more sensitive than HMB 45 [109].

Chondroitin Sulfate Proteoglycan 4 (CSPG4) is involved in tissue development and can be a transmembrane receptor allowing for melanoma motility. Campoli et al. showed that the expression of CSPG4 is seen in 70% of superficial spreading and nodular human melanomas at multiple stages of melanoma progression [94,110].

Tyrosinase is involved in melanin synthesis and is expressed in epidermal melanocytes as well as pigmented portions of the eye including the retina, iris, and ciliary body [97]. Tyrosinase is also expressed in junctional nevi as well as in the junctional zone of compound nevi, with decreasing expression in the deeper areas [111]. In the review done by Ordóñez, he noted that tyrosinase has been seen to also be positive in clear cell sarcomas, pigmented neurofibromas, and a low percentage of angiomyolipomas [97].

PNL2 is a monoclonal antibody that does not have a target antigen known but reacts with normal melanocytes and neutrophils [108]. After Ordóñez performed multiple studies, he concluded that PNL2 is a highly sensitive and specific melanoma marker that is often positive in primary epithelioid melanomas and metastatic melanomas [97,108]. PNL2 has also been reported positive in clear cell sarcomas, renal angiomyolipomas, lymphangioleiomyomatosis, and melanocytic schwanommas [108,112].

MITF, the microphthalmia transcription factor protein, plays a role in the differentiation of neural crest-derived melanocytes, mast cells, osteoclasts, and optic cup-derived retinal pigment epithelium [113]. MITF-M is the melanocyte specific isoform that does the transcription regulation of genes and controls melanogenesis, cell survival, and differentiation [114]. Ordóñez found that the sensitivity and specificity of MITF is lower than other melanoma biomarkers. MITF is similarly expressed in Schwann cells, stromal fibroblasts, dermal scars, and some mesenchymal and neural spindle cell neoplasms, which can easily be mixed up with desmoplastic melanoma [114,115]. MITF lacks specificity, so it is not beneficial for use in differentiating epithelioid melanomas from carcinomas but does have the advantage of being expressed in the nucleus, making the interpretation of IHC easier to read [97].

SOX10 is involved in the embryonic determination of cell fate and is critical in the development and formation of melanocytes [116,117]. SOX10 is a sensitive biomarker for melanocytic tumors that can be expressed in both primary and metastatic melanomas [97,99]. SOX10 stains in a nuclear pattern and is not expressed in dendritic cells, making it more beneficial for lymph node staining [118,119]. SOX10 is not restricted to solely the melanocyte, and it is found in hepatocytes, renal tubular cells, adrenal medullary cells, and the myocardium [120].

Melanocortin 1 Receptor (MC1R) is a melanocyte-stimulating hormone receptor in the GPCR (G protein-coupled receptor) family that controls pigment and plays a large role in the skin phenotype and sensitivity [121]. In two studies reviewed by Ordóñez [120,122], MC1R was present in 100% of the 44 melanomas.

PRAME (preferential expressed antigen in melanoma) is a member of the cancer testis antigen family that has normal expression in the testis, ovaries, adrenals, endometrium, and placenta [123,124]. These proteins encode antigens that are subsequently recognized by T lymphocytes [123]. In a study done by Lezcano et al., they tested 110 melanocytic tumors with ambiguous features by PRAME immunohistochemistry (IHC) and cross-referenced them with fluorescent in situ hybridization (FISH) and single nucleotide polymorphism (SNP) array. They found agreement in PRAME IHC and final diagnostic interpretation in 102/110 samples (92.7%) [125]. In their previous study from 2018, Lezcano found that 88%–94% of non-spindle cell cutaneous melanomas showed nuclear immunoreactivity for PRAME in >75% of sampled cells. In comparison, benign nevi showed PRAME expression in 13.1% of the samples and was present in less than 50% of specimen cells [124]. These findings suggest the use of PRAME in the workup of ambiguous melanocytic lesions [125].

**Figure 10:**
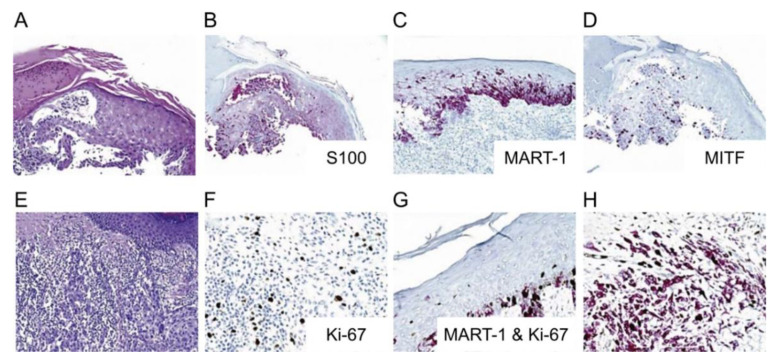
Melanocytic Markers: (Reproduced from “Diagnostic Immunohistochemistry in Cutaneous Neoplasia” by Compton et al., published by Karger Publishers under the Creative Commons CC-BY-NC 3.0 License [132] (**A**) Melanoma in situ, (**B**) S100 stained, (**C**) MART-1 (Melanoma antigen recognized by T cells-Cloned gene) stained, (**D**) MITF (Micropthalmia-associated transcription factor) stained, (**E**,**F**) Ki-67 stain of a melanoma with a tumor infiltrating lymphocytic response (**G**,**H**) Ki-67 and MART-1 stain on a melanoma that shows melanocytic lineage cellular proliferation [132].

**Figure 11:**
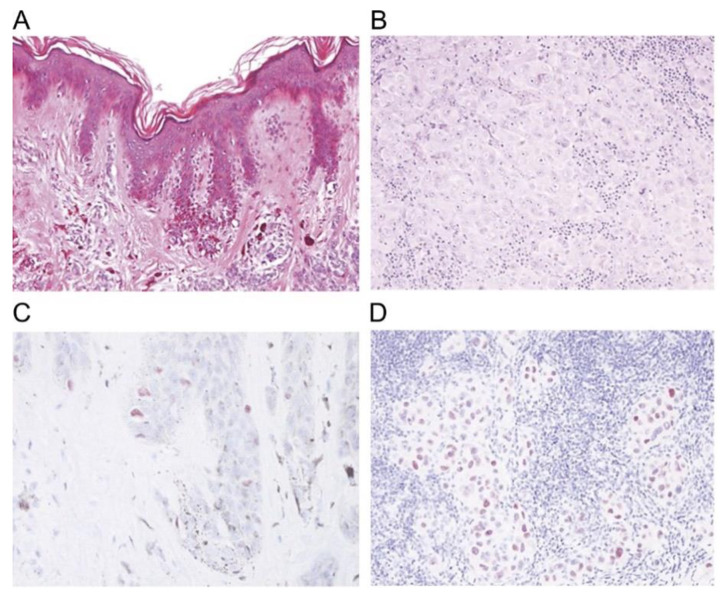
Immunohistochemical stain for PRAME (preferential expressed antigen in melanoma): (Reproduced from “Metastatic PRAME Expressing Juvenile Spitzoid Melanoma on the Buttock” by Muto et al., published by Karger Publishers under the Creative Commons CC-BY-NC 4.0 license, © The Author(s) [133]. (**A**) Histology of primary lesion, (**B**) Histologic findings of a metastatic lymph node, (**C**) PRAME immunohistochemical staining of the primary tumor (**D**) PRAME immunohistochemical staining of a metastatic lymph node [133].

**Figure 12:**
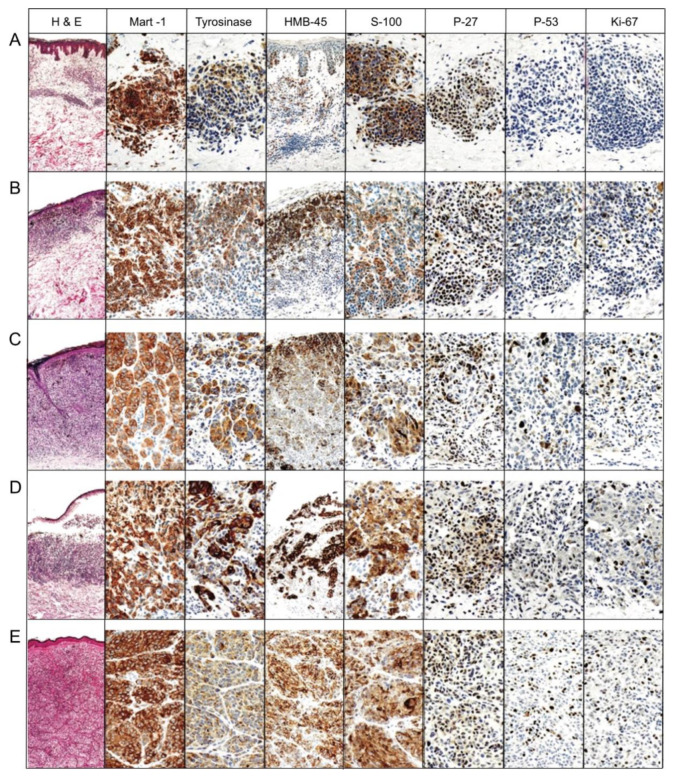
Immunohistochemical stains of five different lesions. Six different stains were applied to five different histologic sections including (**A**) intradermal nevus, (**B**) dysplastic (Clark’s) nevus, (**C**) nevoid melanoma, (**D**) NOS (Not otherwise specified) primary melanoma, (**E**) metastatic melanoma. Stain types included hematoxylin and eosin (H&E), MART-1, Tyrosinase, HMB-45, S-100, P-27, P-53 and K-i67. In melanoma, HMB-45 showed a staining of deeper cells, as compared to the primarily superficial staining that is seen in both benign and dysplastic nevi. The P-53 marker shows positive staining in melanomas but was also positive in dysplastic nevi, while not staining positively in common nevi. Ki-67 demonstrated deeper staining in the bottom of melanoma lesions, while showing positive staining primarily in the junctional melanocytes of the common nevi. With positivity in both melanomas and common nevi, P-27 is a poor distinguishing marker. The following markers were photographed by the original author at 100× the original magnification: Tyrosinase, S-100, p-27, p-53, Ki-67 and MART-1 (Melanoma antigen recognized by T-cells). HMB45 stain was photographed by the original author at 40× of the original magnification. All of the H&E images were photographed by the original author at 20× [128]. Reproduced from Ohsie et al. from the Journal of Cutaneous Pathology with permission from publisher John Wiley and Sons, [128]).

Other studied immunomarkers include pHH3 and p16. Tissue growth is identified when stained for pHH3 and correlates with mitosis specifically by looking at the phosphorylation of histone H3 [95,126]. There is some concern that pHH3 may overestimate mitoses in both melanocytes and nonmelanocytic mitoses in the tissue [95]. The product of the cyclin-dependent kinase inhibitor 2A (*CDKN2A*) gene is p16 protein. In the review by Abbas et al., they found several studies that have shown a decrease in nuclear staining with p16 in melanomas (50%–98% show loss) and that p16 could be used for differentiating melanoma from spitz nevi [95]. Table 2 summarizes each immunohistochemical marker, function, current use, and staining pattern. Figure 10, Figure 11 and Figure 12 illustrate the immunostaining of various histopathology specimens.

### 4.5. Serologic/Molecular Diagnosis

In addition to studying markers within the tissue themselves, current research has shifted toward seeking out melanoma biomarkers within the serum. While LDH (lactate dehydrogenase) is the most widely known serum biomarker in melanoma as a strong prognostic factor [134], its use in diagnosis is limited. Deichmann et al. demonstrated that LDH is the most specific serum biomarker in melanoma with a 92% specificity and 79% sensitivity [135]. LDH is currently the only serum biomarker accepted by the American Joint Committee on Cancer staging system as having a prognostic value for melanoma [98]. Neagu et al. discussed research on microRNA. They showed that miRNA-200c, miRNA-205, and miRNA-23b were downregulated in melanoma, while miR-146a and miR-155 were upregulated [130,136]. Armand-Labit et al. did a study with miR-1246 and miR-185, finding them to be associated with metastatic melanoma. These microRNA biomarkers in the plasma have the potential to serve in early detection of melanoma [130,137].

S100B in the serum has also been seen to correlate with the clinical stage of melanoma according to Fagnart et al. [94,138]. S100B has a direct action on TP53, a known tumor suppressor, and the effect of S100B allows for increased tumor growth in melanoma [21,28]. In a study done by Guo et al., serum S100B was normal in healthy people, and it increased in those with melanoma. In stages I/II, 1.3% of people were found to have elevated levels. In stage III, 8.7% had elevated levels, and in stage IV, 73.9% of patients had elevated levels of serum S100B [139]. Weinstein et al. suggests that S100 is not beneficial in early melanoma detection, but it is better suited for evaluation in patients with advanced disease [98].

Another serologic test possibility on the horizon is the use of genetic screening for the identification and risk stratification of patients based on their likelihood of developing melanoma. This is especially pertinent in patients with conditions that predispose to the development of melanoma such as mutations in *PTEN* (Cowden syndrome), *TP53* (Li Fraumeni syndrome), and *multiple XP genes* (xeroderma pigmentosum) [140]. Other genes including *CDKN2A*, *CDK4*, *BAP1*, *POT1*, *ACD*, *TERF2IP*, and *TERT* are known for their high penetrance as predisposing mutations for melanoma [141]. While *CDKN2A*, *BRCA1* protein, and *CDK4* genes are known susceptibility genes that are considered to be high risk for melanoma, a well-established clinical utility for testing these gene must first be established [12]. The genetic biomarkers are a promising niche that we continue to better understand each year.

## 5. Conclusions

Diagnosing melanoma is still a multistep process, requiring multiple diagnostic biomarkers to be present. Careful visual inspection is the first method of detection for a concerning lesion. Further investigation is either performed through a non-invasive imaging method or through an invasive biopsy with a subsequent histopathology. With the addition of the use of immunohistochemical markers, going one step beyond the cellular morphology, the differentiation of melanoma from its benign counterparts becomes clearer. Following the immunohistochemical analysis, serologic and molecular testing can help confirm melanotic subtypes, determine prognosis, and guide treatment; there is a hope that molecular testing will become a diagnostic method in the future.

The American Cancer Society has current projections for 2020 that show that melanoma continues to increase. In the US alone, 100,350 new melanomas will be diagnosed and 6850 people will die from melanoma [2]. With these rising numbers, prevention and early detection are paramount to curbing this variant of skin cancer. The gold standard for melanoma evaluation has been to interpret an excisional biopsy [142] for its pathological characteristics of cutaneous melanoma such as tumor thickness, ulceration, and mitotic rate [143]. Today, these identifiers can only be determined after the tumor is visually identified, biopsied or surgically removed, and analyzed for its histopathology. With improved methods of detection including the automatic detection of melanoma using image processing of dermoscopy images, more specific morphological features, more specific immunohistochemical stains, and more accurate serologic testing, the field is moving in the right direction.

The main goal of this review was to identify different melanoma diagnosis features that can be used in biomedical sensing tools including imaging modalities for non-invasive or minimally invasive early diagnosis of melanoma, melanoma staging, and melanoma treatment management.

## Figures and Tables

**Table 1:** List of mandatory and additional histopathologic features to be reported by a pathologist.

Mandatory Features [44]	Additional Features [36]
Ulceration	Melanocytes that are more often arranged as solitary predominating over melanocytes in nests
Mitotic rate	Irregular distribution of nests
Regression	Nests of melanocytes varying in size and shape
Lymphovascular Invasion	Slight cytological atypia, namely mild cellular pleomorphism, larger sized cells than normal cells, abundant cytoplasm, and distinct nucleoli
Perineural Invasion	Single cells often extending irregularly far down to the eccrine duct epithelium
Breslow Thickness	Pagetoid spread of melanocytes, ascent of melanocytes in the granular layer
Satellitosis	
Status of Surgical Margin	

**Table 2:** Immunohistochemical biomarkers with their function, current use, and staining pattern.

Immunohistochemical Biomarker	Function	Current Use	Staining Pattern
S100 Protein Family	involved in multiple cellular processes such as cellular growth, cell cycle progression, cellular motility, calcium homeostasis, transcription, and protein phosphorylation [97,98]	highly sensitive (93%–100%) and stains most melanocytic lesions, but lacks specificity [97,127]	nuclear and cytoplasmic, strong and diffuse [97,128]
HMB 45	monoclonal antibody against PMEL17 (gp100), plays role in organizational structure of melanoma [97]	very specific, lower sensitivity (70%–90%) than S100 [97]	cytoplasmic, finely granular [97]
Ki-67	non-histone nuclear protein, proliferation marker [95]	useful in differentiating benign nevi from melanoma [95]	nuclear [95]
Melan A	assists in processing of PMEL17 for the formation of stage II melanosomes [97]	sensitivity is 85%–97% for primary melanoma, sensitivity is 57%–92% for metastatic melanoma, specificity is 95%–100% [97], not expressed in dendritic cells in lymph nodes [97]	cytoplasmic [97]
CSPG4	tissue development and cell motility [94,98]	more sensitive detection for metastatic melanoma than S100B, HMB 45, and MART-1 [94]	tumor cell membrane [129]
Tyrosinase	primary enzyme involved in melanin synthesis [97]	highly specific (97%–100%) [97,98] for primary melanoma [98], expressed in clear cell sarcomas, pigmented neurofibroma, and 20% of angiomyolipomas [97]	cytoplasmic [97]
PNL2	monoclonal antibody with unknown antigen, reacts with neutrophils and melanocytes [97]	positive in 75%–100% of primary metastatic and epithelioid melanomas, can stain positive in PEComas, clear cell sarcoma, and melanocytic schwannomas [97]	cytoplasmic [97]
MITF	neural crest cell differentiation [97]	limited use due to lack of specificity for melanoma but may be useful in an immunohistochemical panel [97]	nuclear [97,128,130]
SOX10	embryonic determination of cell fate [97]	sensitive for primary and metastatic melanomas [97], positive in clear cell sarcomas and peripheral nerve sheath tumors [97]	nuclear [97]
MC1R	G protein-coupled receptor family that controls pigment and plays role in skin phenotype and sensitivity [97]	sensitive for melanoma, not restricted to only melanocytes. Also present in neurons, hepatocyte, renal tubular cells, adrenal medullary cells, and myocardial cells [97,120]	cell surface and intracellular expression [120]
PRAME	part of the cancer testis antigen family, antigen recognition by T lymphocytes [123]	Stains positive in >75% of cells present in non-spindle cell cutaneous melanoma and is positive in 88%–94% of non-spindle cell cutaneous melanoma cases [124]	nuclear, diffuse reactivity [124]
pHH3	correlates with mitosis looking for phosphorylation of histone H3 [95]	may overestimate mitoses due to melanocyte and non-melanocyte melanomas [95]	nuclear [131]
p16	protein product of *CDKN2A* gene [95]	50%–98% of melanomas show loss of nuclear staining [95]	nuclear, decreased in melanomas [95]
